# Supplementary material for: Altered microRNA Transcriptome in Cultured Human Liver Cells upon Infection with Ebola Virus
Source: Int J Mol Sci. 2021 Apr 6;22(7):3792. doi: 10.3390/ijms22073792 (PMC8038836; doi:10.3390/ijms22073792)
Supplement: Supplementary file 1 [file ijms-22-03792-s001.zip › Supplementary_File/C_ GO_Analysis_Results/16-30nt_go_Reston-24h-Huh7_vs_Control-24h-Huh7_up.mature_mirna_targets/CC_Pvalue_tree.pdf]

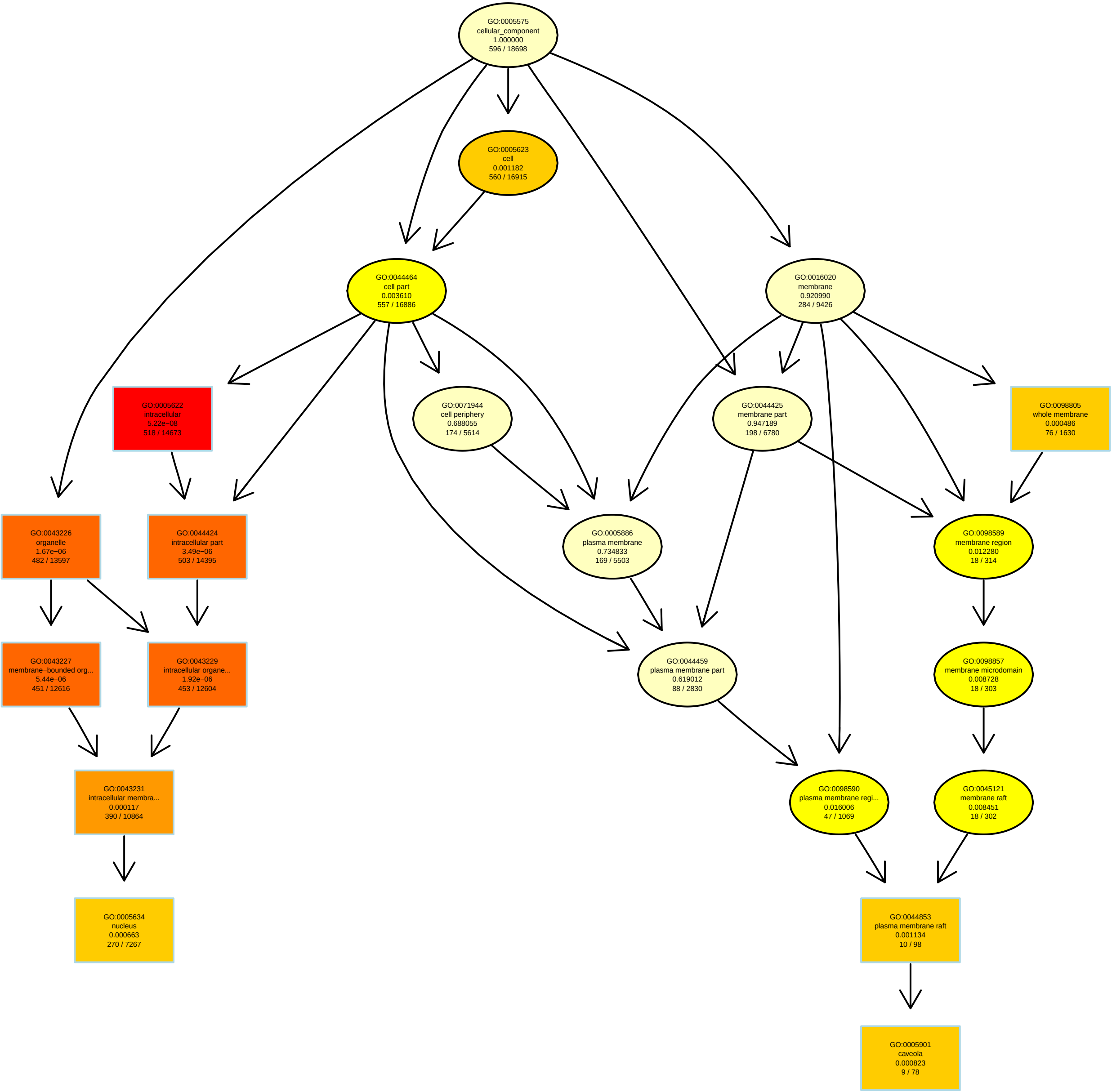
GO:0005575
cellular_component
1.000000
596 / 18698
GO:0005623
cell
0.001182
560 / 16915
GO:0044464
cell part
0.003610
557 / 16886
GO:0016020
membrane
0.920990
284 / 9426
GO:0005622
intracellular
5.22e−08
518 / 14673
GO:0071944
cell periphery
0.688055
174 / 5614
GO:0044425
membrane part
0.947189
198 / 6780
GO:0098805
whole membrane
0.000486
76 / 1630
GO:0043226
organelle
1.67e−06
482 / 13597
GO:0044424
intracellular part
3.49e−06
503 / 14395
GO:0005886
plasma membrane
0.734833
169 / 5503
GO:0098589
membrane region
0.012280
18 / 314
GO:0043227
membrane−bounded org...
5.44e−06
451 / 12616
GO:0043229
intracellular organe...
1.92e−06
453 / 12604
GO:0044459
plasma membrane part
0.619012
88 / 2830
GO:0098857
membrane microdomain
0.008728
18 / 303
GO:0043231
intracellular membra...
0.000117
390 / 10864
GO:0098590
plasma membrane regi...
0.016006
47 / 1069
GO:0045121
membrane raft
0.008451
18 / 302
GO:0005634
nucleus
0.000663
270 / 7267
GO:0044853
plasma membrane raft
0.001134
10 / 98
GO:0005901
caveola
0.000823
9 / 78
